# Spontaneous resolution of a unilateral cataract in an adult: A case report

**DOI:** 10.1097/MD.0000000000029466

**Published:** 2022-06-24

**Authors:** Woo Seok Choe, Moosang Kim, Tae Gi Kim

**Affiliations:** a Department of Ophthalmology, Kyung Hee University Hospital at Gangdong, Kyung Hee University, Seoul, Republic of Korea; b Dabom EYE Clinic, Yangju-si, Gyeonggi-do, Republic of Korea.

**Keywords:** cataract, blood glucose, posterior subcapsular cataract, spontaneous regression

## Abstract

**Rationale:**

Cataracts are a disease that is usually caused by aging and involve the irreversible degeneration of the lens material. On the other hand, transient cataracts have also been reported, mainly due to systemic hyperglycemia, which often occurs bilaterally. However, reports of the spontaneous regression of unilaterally occurring cataracts in patients with normal blood glucose levels are rare. Here, we report a rare case of spontaneous regression of unilateral posterior subcapsular cataracts in an adult with normal blood glucose levels.

**Patient concerns:**

A 42-year-old woman presented with distorted vision in her right eye. The patient was taking medication for diabetes, and her blood sugar level was well-controlled.

**Diagnosis:**

Upon examination, her uncorrected visual acuity and best-corrected visual acuity were 20/70 in her right eye. Slit lamp microscopy revealed fine, feathery, and streak-like posterior subcapsular opacities. Color fundus photography revealed a star-shaped shadow due to the cataract, and no diabetic retinopathy was observed. Her two hour postprandial glucose level was 115 mg/dL. The patient was diagnosed with posterior subcapsular cataracts, and cataract surgery was planned. The patient was scheduled to visit the clinic again after seven days.

**Interventions:**

Close observation for one week without any intervention.

**Outcomes:**

After one week, most of the posterior subcapsular opacities disappeared, and the uncorrected visual acuity and best-corrected visual acuity in the right eye improved to 20/40 and 20/30, respectively.

**Lessons:**

This case report demonstrates that unilateral posterior subcapsular cataracts may spontaneously regress in patients with normal blood glucose levels. Therefore, it is important to check whether cataracts improve spontaneously through short-term close follow-up before planning cataract surgery to avoid unnecessary surgery.

## 1 Introduction

Cataract, the loss of transparency of the lens, are one of the most common causes of visual loss.^[1]^ Cataract can have various causes, the main risk factor is aging. In general, lens clouding is irreversible and progressive and causes a steady decline in vision. However, in rare cases, spontaneous cataracts regression has been reported.^[2–15]^ Among the various cataract types, most spontaneous resolution has been reported in posterior subcapsular cataract (PSC) that show dynamic changes unlike other types of cataracts.^[16]^ In PSC, vision loss can occur early, because the opacity is located at the nodal point of the lens.

Spontaneous resolution of cataracts has been reported due to local causes, such as intraocular surgery, trauma, and yttrium aluminum garnet laser iridotomy.^[2–7]^ Furthermore, the spontaneous regression of cataracts has also been reported due to systemic causes, such as uncontrolled hyperglycemia.^[8–15]^ The mechanism of transient cataract occurrence in hyperglycemia is presumed to be due to osmotic stress. Furthermore, most cataracts occur in both eyes, and when hyperglycemia improves, cataracts also improve.^[2–15]^ However, spontaneous resolution of unilateral cataracts in patients with well-controlled diabetes is very rare. In general, even if there is an underlying disease such as diabetes, if no major abnormalities are observed in the current examination, cataract surgery is often planned immediately without follow-up, which may lead to unnecessary cataract surgery.

Herein, we report an unusual case of unilateral transient cataracts in a 42-year-old woman with type 2 diabetes mellitus with good glycemic control and review the available literature.

Informed written consent was obtained from the patient for publication of this case report and accompanying images. No ethical approval was obtained, because this study was a retrospective case report and did not involve a prospective evaluation.

## 2 Case report

A 42-year-old woman presented to our clinic with complaints of distorted vision in her right eye. A review of her medical history revealed that she had diabetes and was taking oral hypoglycemic agents. She said that her blood glucose was well regulated and that, to date, she had not been diagnosed with diabetes-related complications. A two hour postprandial glucose test performed at our clinic was 115 mg/dL.

**Figure 1:**
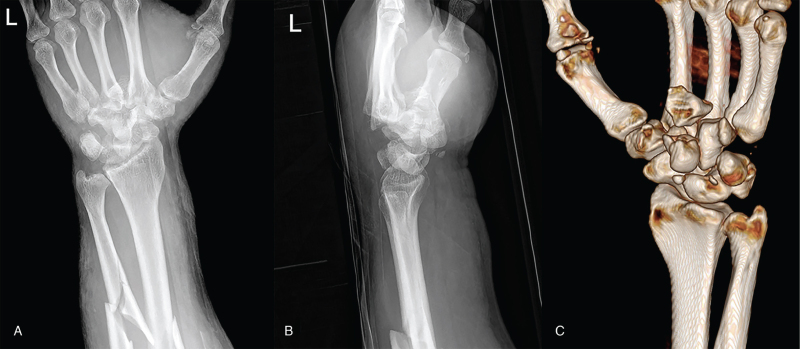
Anterior and fundus photography at the patient's first visit. (A) Retroillumination revealed a dense rosette posterior subcapsular cataract in the right eye. (B) Fundus photography revealed a feathery streak-like shadow due to posterior subcapsular cataract, and no diabetic retinopathy was evident in the right eye.

On ophthalmic examination, her uncorrected visual acuity (UCVA) and best-corrected visual acuity (BCVA) were 20/70 in the right eye and the UCVA was 20/30 and BCVA was 20/20 in the left eye. Automated refraction revealed −4.25 diopters (D) of spherical equivalent (SE) in the right and −2.00 D of SE in the left eye. Intraocular pressure, measured using a non-contact tonometer, was 13 mmHg in the right eye and 11 mmHg in the left eye. Through a widely dilated pupil, the slit-lamp examination showed a PSC with fine, feathery-like opacities that radiated towards the periphery in the right eye (Fig. 1A). Color fundus photography revealed no other retinal abnormalities, including diabetic retinopathy, except for a star-shaped shadow caused by posterior subcapsular opacity (Fig. 1B). Cataract surgery on the right eye was planned, and an appointment was made to return for seven days for biometry measurements.

**Figure 2:**
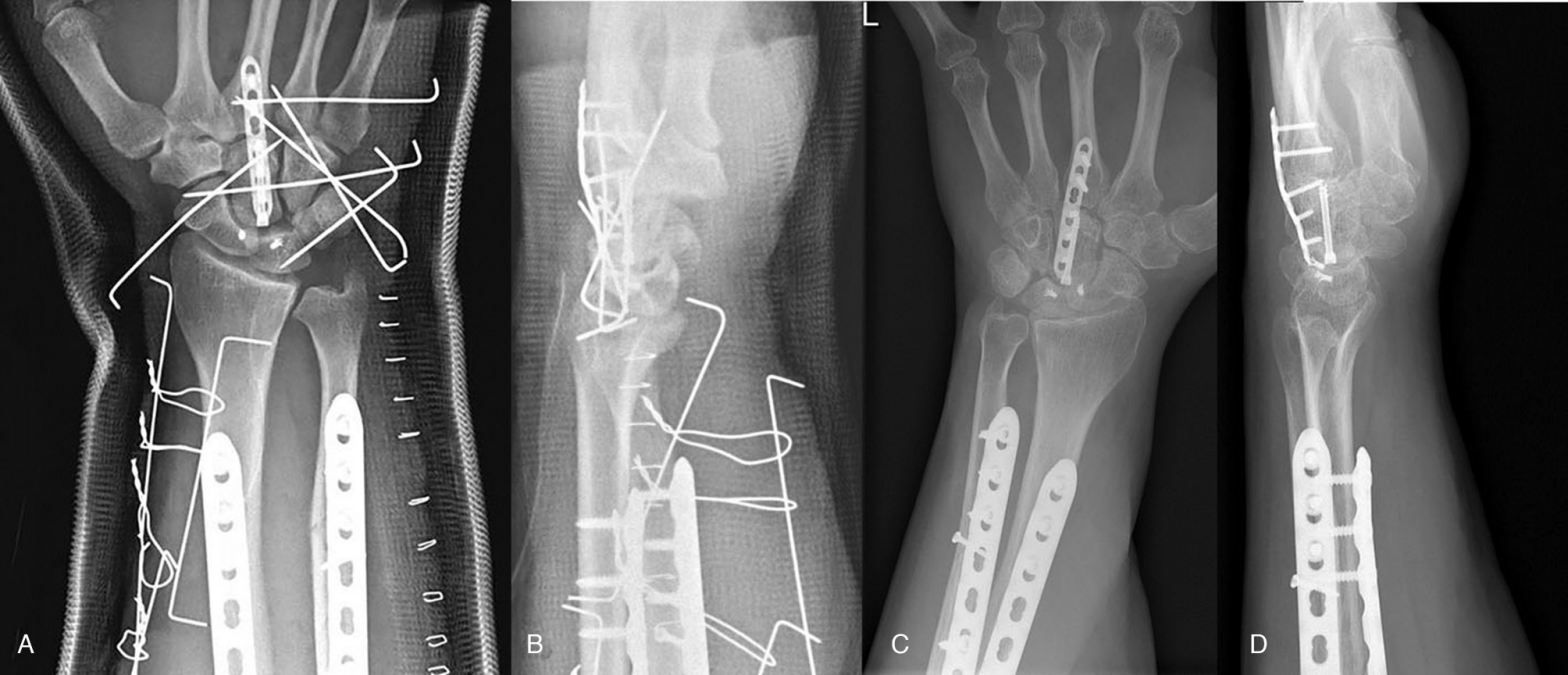
Regression of the posterior subcapsular cataract one week after initial presentation in the right eye. (A) External examination of the lens revealed resolution of the most of posterior subcapsular opacity. (B) Color fundus photography revealed that the range and density of the cataract shadow reduced.

At the one week follow-up, we observed that the size of the PSC had decreased (Fig. 2A), and color fundus photography revealed that the star-shaped shadow due to PSC had decreased. (Fig. 2B). Her right visual acuity improved to 20/40 and 20/30 for the UCVA and BCVA, respectively. Her SE was −4.25 D in the right eye and −1.75 D in the left eye, with no significant difference from seven days prior. Although her visual acuity improved, the center of the posterior subcapsular opacity was located at the nodal point, and the patient still experienced decreased visual acuity. Therefore, cataract surgery was performed in the right eye.

## 3 Discussion

The spontaneous regression of cataracts is rare but has been reported previously, mostly of cataracts caused by conditions unrelated to senile change, such as ocular trauma, surgery, laser, and hyperglycemia.^[2–15]^ The cases of spontaneous cataract regression reported to date are summarized in Table 1, following a PubMed search.

**Table 1:** Summary of published cases of transient cataracts.

Authors	Year	Number of patients	Age (y)/Sex	Cataract type	Etiology	Direct cause	Bilaterality	Follow-up
Su et al^[2]^	2021	1	44/M	PSC	Trauma	Intraocular Foreign body	Unilateral	Resolved after 3 months
Zhang et al^[3]^	2020	1	13/M	PSC	Trauma	Open globe injury	Unilateral	Resolved after 9 months
Bansal and Fenerty^[4]^	2020	1	54/M	PSC	Trauma	After Nd:YAG laser	Unilateral	Resolved after 4 months
Yang et al^[5]^	2018	1	20/M	ASC	Trauma	Distilled water	Unilateral	Resolved after 1 month
Park^[8]^	2017	1	42/M	PSC	Diabetes	After hyperglycemia control	Bilateral	Resolved after 1 month
Jin et al^[9]^	2012	2	11/F9/F	PSC	Diabetes	Hyperglycemia	Bilateral	Resolved after 5 and 3 months
Rofagha et al^[6]^	2008	1	49/M	PSC	Trauma	Intralenticular foreign body	Unilateral	Resolved after 1 month
Ramkumar and Basti^[10]^	2008	1	58/F	PSC	Diabetes	Hyperglycemia	Unilateral	Resolved after 1 month
Trindade^[11]^	2007	1	13/M	PSC	Diabetes	Hyperglycemia	Bilateral	Resolved after 2 weeks
Forbes et al^[17]^	2004	1	0/M	PSC	Unkown(Trauma)	Unkown(Birth injury)	Unilateral	Resolved after 2 months
Sharma and Vasavada^[12]^	2001	1	62/M	PSC	Diabetes	Hyperglycemia	Bilateral	Resolved after 5 weeks
Butler^[13]^	1994	1	52/M	PSC	Diabetes	Hyperglycemia	Bilateral	Resolved after 7 weeks
Saito et al^[14]^	1993	4	53, 28/F53, 45/M	PSC	Diabetes	Hyperglycemia	Bilateral	Resolved after 4 to 14 weeks
Yap and Buettner^[7]^	1992	1	19/F	PSC	Trauma	Intraocular Foreign body	Unilateral	Resolved after 3 years
Epstein^[15]^	1976	1	54/M	PSC	Diabetes	Hyperglycemia	Unilateral	Resolved after 1 week
Choe et al	2022	1	42/F	PSC	Unkown	Unkown	Unilateral	Resolved after 1 week

ASC = anterior subcapsular cataract, Nd:YAG = neodymium-doped yttrium aluminum garnet, PSC = posterior subcapsular cataract.

The spontaneous regression of traumatic cataracts is mainly seen in cases of intraocular foreign bodies, penetrating injury, yttrium aluminum garnet laser, and cataracts caused by distilled water.^[2–7]^ When lens integrity is compromised due to direct damage to the lenticular capsule, cataracts can occur due to aqueous humor influx into the lens lamella and denaturation of the lens protein.^[16]^ However, lenticular capsule damage is limited and can heal without progressive cataract formation in rare cases, leading to resolution.^[6]^

On the other hand, spontaneous improvement in non-traumatic cataract occurs mostly in cases where the cataract is caused by uncontrolled acute hyperglycemia. The cataract reverses after good glycemic control without fluctuations. When hyperglycemia occurs, sorbitol is deposited on the lens fibers, which creates an osmotic force and leads to water influx from the aqueous humor into the lens.^[15]^ When blood sugar is well controlled, the lens expels water from the lens by pumping and metabolizing sorbitol. With this mechanism, the opacity of the lens can be reversibly improved.^[15]^ Cataracts caused by hyperglycemia generally occur bilaterally and have a snowflake cataract pattern with subcapsular vacuoles, as in this case. In addition to lens opacity, refractive errors, such as hyperopic shift through lens swelling, are common. However, in our patient, cataracts occurred in only one eye, blood glucose levels were within normal values, and no hyperopic shift was observed.

Cataracts with spontaneous regression in adult patients with normal serum glucose levels without a history of trauma are rare and have been infrequently reported in the literature. In 2004, Fores et al reported a case of unilateral PSC in a healthy infant that regressed after two months of observation.^[17]^ Although the cause of cataracts in infants remains uncertain, it has been speculated to occur through posterior capsular rupture at birth, with hydration of the cortical material.

As shown in Table 1, most cataracts that show spontaneous regression are in the form of PSC. The mechanism cannot be clearly explained, but it can be assumed that PSC has a dynamic nature that differs from other types of cataracts. PSC is a type of cataract in which opacity occurs in the lens capsule of the posterior pole. Pathologically, PSC occurs when metaplastic cells located in the lens equator move to the posterior pole.^[18]^ Unlike nuclear cataracts, which cause irreversible protein degradation, PSC are known to occur mainly due to disturbances in the ion pump or alteration of metabolic enzyme activity.^[18]^ Therefore, PSC can have the characteristics of a dynamic process, which might partially explain the spontaneous PSC regression in our patient. Neumayer et al reported the changes and disappearance of lens vacuoles for four weeks in patients with PSC.^[19]^ In addition, Magno et al reported that PSC progressed in 8% of patients and regressed in 4% of patients during a six-month follow-up.^[20]^ It is difficult to explain why cataracts developed only in the right eye. However, since PSC can change naturally, even at the focal location of the lens, it occurred only in the right eye. Spontaneous regression was thought to be possible in our patient.

## 4 Conclusion

In conclusion, transient cataracts can occur even in the absence of systemic hyperglycemia. Such cataracts can occur unilaterally, may not be accompanied by hyperopic shift, and can improve after seven days of observation. Therefore, before deciding on cataract surgery for patients with PSC, awareness of the potential reversibility of this type of cataract is crucial to protect patients from unnecessary surgery.

## Author contributions

**Conceptualization:** Moosang Kim, Tae Gi Kim, Woo Seok Choe.

**Data curation:** Moosang Kim, Tae Gi Kim, Woo Seok Choe.

**Formal analysis:** Moosang Kim, Tae Gi Kim, Woo Seok Choe.

**Investigation:** Tae Gi Kim.

**Methodology:** Moosang Kim.

**Project administration:** Moosang Kim, Tae Gi Kim.

**Resources:** Moosang Kim, Tae Gi Kim, Woo Seok Choe.

**Supervision:** Moosang Kim, Tae Gi Kim, Woo Seok Choe.

**Validation:** Woo Seok Choe.

**Visualization:** Tae Gi Kim, Woo Seok Choe.

**Writing – original draft:** Tae Gi Kim, Woo Seok Choe.

**Writing – review & editing:** Tae Gi Kim, Woo Seok Choe.
