# Spontaneous resolution of a unilateral cataract in an adult: A case report: Erratum

**DOI:** 10.1097/MD.0000000000030549

**Published:** 2022-09-02

In the article, “Spontaneous resolution of a unilateral cataract in an adult: A case report”,^[1]^ which appears in Volume 101, Issue 25 of *Medicine*, the incorrect images appeared as Figure 1 and 2. They have now been corrected to the appropriate images.
